# Acute effects of MDMA and LSD co-administration in a double-blind placebo-controlled study in healthy participants

**DOI:** 10.1038/s41386-023-01609-0

**Published:** 2023-05-31

**Authors:** Isabelle Straumann, Laura Ley, Friederike Holze, Anna M. Becker, Aaron Klaiber, Kathrin Wey, Urs Duthaler, Nimmy Varghese, Anne Eckert, Matthias E. Liechti

**Affiliations:** 1 Clinical Pharmacology and Toxicology, Department of Biomedicine and Department of Clinical Research, University Hospital Basel, Basel, Switzerland; 2 Department of Pharmaceutical Sciences, University of Basel, Basel, Switzerland; 3 Psychiatric University Hospital, University of Basel, Basel, Switzerland; 4 Transfaculty Research Platform Molecular and Cognitive Neuroscience, University of Basel, Basel, Switzerland

**Keywords:** Translational research, Neurophysiology

## Abstract

There is renewed interest in the use of lysergic acid diethylamide (LSD) in psychiatric research and practice. Although acute subjective effects of LSD are mostly positive, negative subjective effects, including anxiety, may occur. The induction of overall positive acute subjective effects is desired in psychedelic-assisted therapy because positive acute experiences are associated with greater therapeutic long-term benefits. 3,4-Methylenedioxymethamphetamine (MDMA) produces marked positive subjective effects and is used recreationally with LSD, known as “candyflipping.” The present study investigated whether the co-administration of MDMA can be used to augment acute subjective effects of LSD. We used a double-blind, randomized, placebo-controlled, crossover design with 24 healthy subjects (12 women, 12 men) to compare the co-administration of MDMA (100 mg) and LSD (100 µg) with MDMA and LSD administration alone and placebo. Outcome measures included subjective, autonomic, and endocrine effects and pharmacokinetics. MDMA co-administration with LSD did not change the quality of acute subjective effects compared with LSD alone. However, acute subjective effects lasted longer after LSD + MDMA co-administration compared with LSD and MDMA alone, consistent with higher plasma concentrations of LSD (C_max_ and area under the curve) and a longer plasma elimination half-life of LSD when MDMA was co-administered. The LSD + MDMA combination increased blood pressure, heart rate, and pupil size more than LSD alone. Both MDMA alone and the LSD + MDMA combination increased oxytocin levels more than LSD alone. Overall, the co-administration of MDMA (100 mg) did not improve acute effects or the safety profile of LSD (100 µg). The combined use of MDMA and LSD is unlikely to provide relevant benefits over LSD alone in psychedelic-assisted therapy. Trial registration: ClinicalTrials.gov identifier: NCT04516902.

## Introduction

Lysergic acid diethylamide (LSD) is a classic serotonergic psychedelic that is widely used recreationally and increasingly investigated in patients who suffer from psychiatric conditions, such as anxiety and depression [1, 2]. LSD acutely produces mostly positive experiences of alterations of consciousness but may also produce negative subjective effects, including acute anxiety [3–8]. Acute negative psychological effects are also considered the main risk of psychedelic substance use in humans [9]. Clinical trials showed that positive psychedelic-induced experiences are associated with more positive long-term therapeutic improvements in patients in psychedelic-assisted therapy [1, 10–13]. Additionally, low ratings of acute anxiety induced by a psychedelic predicted positive long-term clinical outcomes in patients [10]. Thus, the induction of a positive acute psychedelic experience may be desirable to enhance treatment outcome, although challenging experiences may also have therapeutic potential [14, 15].

3,4-Methylenedioxymethamphetamine (MDMA) is investigated in MDMA-assisted therapy [16]. MDMA acutely induces mostly positive subjective effects, including increases in well-being, empathy, trust, and closeness to others [3, 17–19]. The combined administration of MDMA and LSD is known as “candyflipping” among recreational substance users [20–24] and reportedly induces synergistic acute positive mood effects [24]. However, no controlled study has investigated the combined administration of MDMA and LSD. Therefore, the present study investigated whether MDMA can be used to optimize the acute effects profile of LSD by inducing more positive mood and less anxiety compared with LSD alone.

The primary hypothesis was that the co-administration of MDMA and LSD results in higher acute “good drug effects,” well-being, openness, and trust and lower “bad drug effects” and anxiety compared with LSD administration alone.

## Methods and materials

### Study design

The study used a double-blind, placebo-controlled, crossover design with four experimental test sessions to investigate responses to (*i*) placebo, (*ii*) 100 mg MDMA, (*iii*) 100 µg LSD, and (*iv*) 100 µg LSD + 100 mg MDMA. Block randomization was used with counter-balanced treatment order. The washout periods between sessions were at least 10 days. The study was conducted in accordance with the Declaration of Helsinki and International Conference on Harmonization Guidelines in Good Clinical Practice and approved by the Ethics Committee of Northwest Switzerland (EKNZ) and Swiss Federal Office for Public Health. The study was registered at ClinicalTrials.gov (NCT04516902).

### Participants

Twenty-four healthy participants (12 men and 12 women; mean age ± SD: 30 ± 7 years; range: 25–54 years) were recruited by word of mouth or from a pool of volunteers who had contacted our research group because they were interested in participating in a clinical trial on psychedelics. All of the subjects provided written informed consent and were paid for their participation. Exclusion criteria were age <25 years or >65 years, pregnancy (urine pregnancy test at screening and before each test session), personal or family (first-degree relative) history of major psychiatric disorders (assessed by the Semi-structured Clinical Interview for *Diagnostic and Statistical Manual of Mental Disorders*, 4th edition, Axis I disorders by a trained psychologist), the use of medications (e.g., antidepressants, antipsychotics, and sedatives) that may interfere with the study medications, chronic or acute physical illness (e.g., abnormal physical exam, electrocardiogram, or hematological and chemical blood analyses), tobacco smoking (>10 cigarettes/day), lifetime prevalence of hallucinogens or MDMA use >20 times, illicit drug use within the last 2 months (except for Δ^9^-tetrahydrocannabinol), and illicit drug use during the study period (determined by urine drug tests). The participants were asked to consume no more than 20 standard alcoholic drinks/week and have no more than one drink on the day before the test sessions. Twelve participants had previously used a psychedelic, including LSD (6 participants, 1–3 times), psilocybin (9 participants, 1–2 times), *N,N*-dimethyltryptamine (DMT; one participant, one time), and mescaline (one participant, 1 time). Nine participants had used MDMA (1–13 times), 12 participants had used a stimulant, including methylphenidate (5 participants, 1–10 times), amphetamine (3 participants, 1–7 times), and cocaine (4 participants, 2–10 times), one participant had used 4-bromo-2,5-dimethoxyphenethylamine (2C-B; 1 time), and one participant had used ketamine (1 time). Four participants had never used any illicit drugs with the exception of cannabis.

### Study drugs

LSD base (Lipomed AG, Arlesheim, Switzerland) was administered as an oral solution that was produced according to good manufacturing practice in units that contained 100 µg LSD base in 1 ml of 96% ethanol [25]. The exact analytically confirmed LSD base content (mean ± SD) was 92.5 ± 1.89 µg (*n* = 10 samples). Placebo consisted of identical units that were filled with ethanol only. MDMA (ReseaChem, Burgdorf, Switzerland) was administered in opaque capsules that contained a 25 mg dose of MDMA hydrochloride and an exact analytically confirmed actual MDMA content of 25.40 ± 0.48 mg (*n* = 9 samples). Placebo consisted of identical opaque capsules that were filled with mannitol. A double-dummy method was used. The subjects received four capsules and one solution in each session: (*i*) four placebo capsules and one placebo solution, (*ii*) four 25 mg MDMA capsules and one placebo solution, (*iii*) four placebo capsules and one 100 µg LSD solution, and (*iv*) four 25 mg MDMA capsules and one 100 µg LSD solution. Then, 2.5 h after administration, at the end of each session, and at the end of the study, the participants guessed their treatment assignment to evaluate blinding.

### Study procedures

The study included a screening visit, four 13-h test sessions with follow-up measurements 24 h after drug intake, and an end-of-study visit which took place on average 31 days after the last test session. Test days were separated by at least 10 days. The sessions were conducted in a calm hospital room. Only one research subject and one investigator were present during each test session. The test sessions began at 8:00 AM. A urine sample was taken to verify abstinence from drugs of abuse, and a urine pregnancy test was performed in women. The subjects then underwent baseline measurements. A standardized breakfast (two croissants) was served. Substances were administered at 9:00 AM. The outcome measures were repeatedly assessed for 12 h. Standardized lunches and dinners were served at 1:30 PM and 6:00 PM, respectively. The subjects were never alone during the acute effect phase. The subjects were sent home at 9:15 PM and returned the next day for follow-up measurements at 9:00 AM.

### Subjective drug effects and effect durations

Subjective effects were assessed repeatedly using visual analog scales (VASs) [3, 6] 0.5 h before and 0, 0.5, 1, 1.5, 2, 2.5, 3, 3.5, 4, 5, 6, 7, 8, 9, 10, 11, 12, and 24 h after drug administration. The Adjective Mood Rating Scale (AMRS) [26] was used 0.5 h before and 3, 6, 9, 12, and 24 h after drug administration. The 5 Dimensions of Altered States of Consciousness (5D-ASC) scale [27, 28] was used as the primary outcome measure and was administered 12 h after drug administration to retrospectively rate peak drug effects. Mystical experiences were assessed 12 h after drug administration using the States of Consciousness Questionnaire (SOCQ) [29, 30] that includes the 43-item Mystical Effects Questionnaire (MEQ43) [29], 30-item Mystical Effects Questionnaire (MEQ30) [31], and subscales for “aesthetic experience,” “connectedness,” “distressing experience,” and negative “nadir” effects. Subjective effect measurements are described in detail in the Supplementary Methods online.

The time to onset, time to maximal effect, time to offset, and effect duration were assessed in Phoenix WinNonlin 8.3 (Certara, Princeton, NJ, USA) using the “any drug effect” VAS effect-time plots and an onset/offset threshold of 10% of the maximum individual response as described previously in detail [7, 25].

### Autonomic and adverse effects

Blood pressure, heart rate, and tympanic body temperature were repeatedly measured at baseline and 0, 0.5, 1, 1.5, 2, 2.5, 3, 3.5, 4, 5, 6, 7, 8, 9, 10, 11, 12, and 24 h after drug administration [32]. Pupil size was assessed at baseline and 1, 2.5, 4, 7, 11, and 24 h after drug administration [6]. Adverse effects were assessed 0.5 h before and 12 and 24 h after drug administration using the List of Complaints [33].

### Circulating oxytocin and brain-derived neurotrophic factor

Plasma concentrations of oxytocin were measured before and 1.5, 3, and 6 h after drug administration and were determined as previously described [3, 6, 7, 34]. Serum BDNF levels were measured at baseline and 3, 6, 9, 12, and 24 h after drug administration (Supplementary Methods).

### Plasma LSD and MDMA concentrations

Plasma concentrations of LSD and MDMA and their metabolites were measured before and 0.5, 1, 1.5, 2, 2.5, 3, 3.5, 4, 5, 6, 7, 8, 9, 10, 11, 12, and 24 h after drug administration. Blood was collected into lithium heparin tubes. The blood samples were immediately centrifuged, and the plasma was subsequently stored at −80°C until analysis. Plasma concentrations of LSD and its metabolite 2-oxo-3-hydroxy-LSD (O-H-LSD) were determined by ultra-high-performance liquid chromatography tandem mass spectrometry with a lower limit of quantification of 10 pg/ml [25].

MDMA and its metabolites 3,4-methylenedioxyamphetamine (MDA) and 4-hydroxy-3-methoxymethamphetamine (HMMA) were analyzed in human plasma using high-performance liquid chromatography tandem mass spectrometry as previously described. HMMA concentration was determined after enzymatic deglucuronidation [35].

### Pharmacokinetic analyses

Pharmacokinetic parameters were estimated using non-compartmental methods as described previously [25]. Analyses were conducted using Phoenix WinNonlin 8.3 (Certara, Princeton, NJ, USA).

### Data analysis

Peak (E_max_ and/or E_min_) or peak change from baseline (ΔE_max_) values were determined for repeated measures. The values were then analyzed using repeated-measures analysis of variance (ANOVA), with drug as the within-subjects factor, followed by the Tukey *post hoc* tests using R 4.2.1 software (RStudio, PBC, Boston, MA, USA) and Statistica 12 software (StatSoft, Tulsa, OK, USA). The criterion for significance was *p* < 0.05.

## Results

### Subjective drug effects

**Fig. 1:**
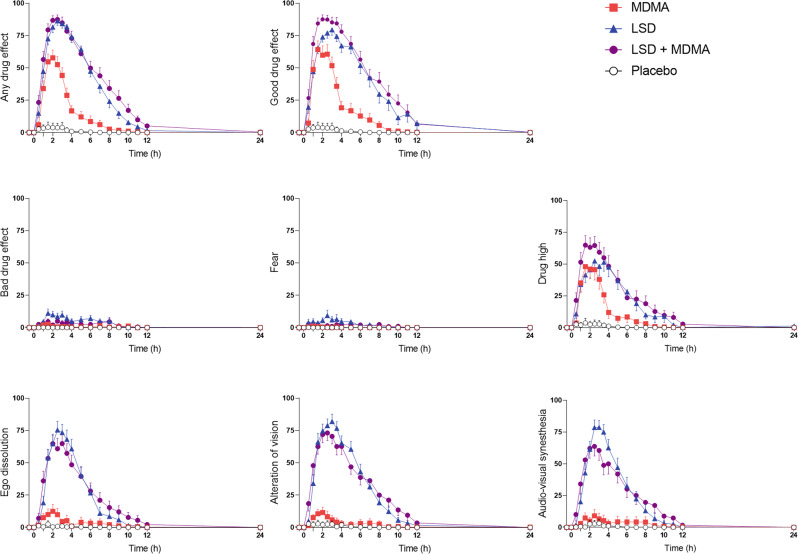
Acute subjective effects of 100 µg lysergic acid diethylamide (LSD), 100 mg 3,4-methylenedioxymethamphetamine (MDMA), and the LSD + MDMA combination over time on the Visual Analog Scale (VAS). LSD and the LSD + MDMA combination produced comparable subjective effects with no significant differences in E_max_ values (Table 1). However, the co-administration of MDMA and LSD prolonged the psychedelic experience compared with LSD alone (Supplementary Table S5). Overall, effects of LSD and LSD + MDMA were significantly stronger and longer compared with MDMA alone. There was no significant difference in peak “drug high” between the substances alone and the combination. The substances were administered at *t* = 0 h. The data are expressed as the mean ± SEM percentage of maximally possible scores in 24 subjects. The corresponding maximal responses and statistics are shown in Table 1.

**Fig. 2:**
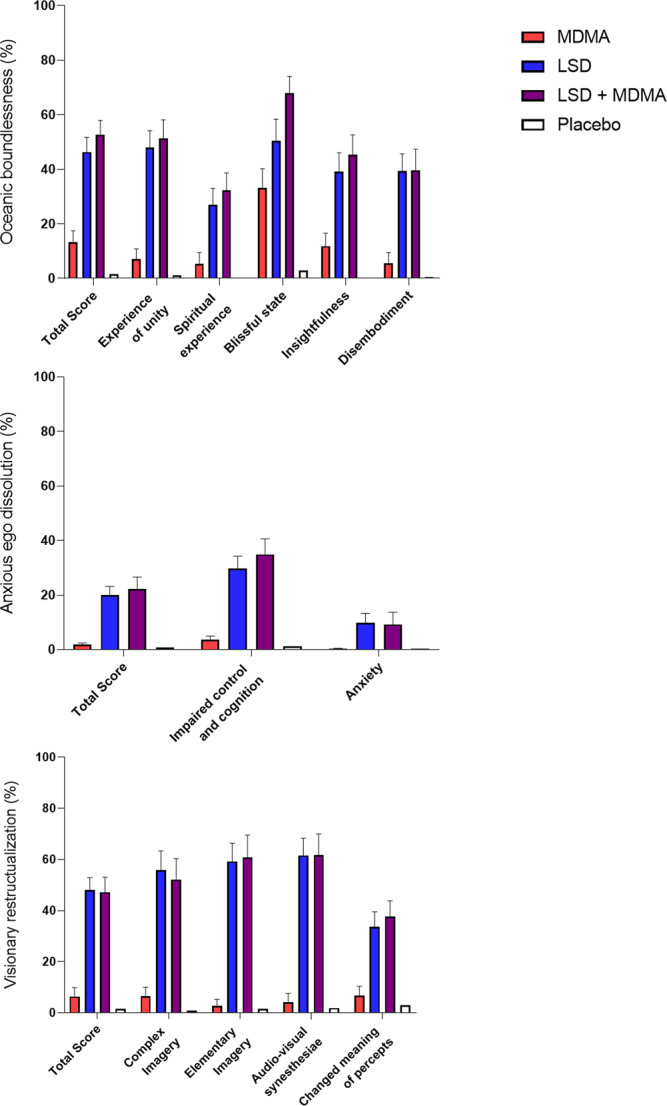
Acute mystical-type experiences on the 5 Dimensions of Altered States of Consciousness (5D-ASC) scale. The combination of LSD (100 µg) and MDMA (100 mg) induced comparable effects to LSD (100 µg) alone. MDMA (100 mg) alone only significantly induced mystical-type effects on the lower-order “blissful state” scale. The data are expressed as the mean ± SEM percentage of maximally possible scale scores in 24 subjects. Statistics are shown in Supplementary Table S2.

Subjective effects over time on the VAS are shown in Fig. 1 and Supplementary Fig. S1. Statistics are summarized in Table 1 and Supplementary Table S1. Alteration of mind and mystical-type effects are shown in Fig. 2 and Supplementary Fig. S2. Statistics are summarized in Supplementary Tables S2 and S3. Effects on mood over time on the AMRS are shown in Supplementary Fig. S3. The corresponding peak responses and statistics are presented in Supplementary Table S4. Characteristics of subjective responses are shown in Supplementary Table S5.

**Table 1:** Mean values and statistics for the acute effects of LSD, MDMA, LSD + MDMA and placebo, *N* = 24.

		Placebo	MDMA	LSD	LSD + MDMA	*F*_3, 69_	*P*=	Pla − MDMA	Pla − LSD	Pla − LSD + MDMA	MDMA −LSD	MDMA − LSD + MDMA	LSD − LSD + MDMA
		Mean ± SEM	Mean ± SEM	Mean ± SEM	Mean ± SEM								
Visual Analog Scale (VAS, %max)													
Unidirectional Scales (0–100)													
Any drug effect	ΔE_max_	5.3 ± 4.0	64 ± 5.6	90 ± 3.1	93 ± 2.6	145	<0.001	***	***	***	***	***	NS
Good drug effect	ΔE_max_	5.1 ± 4.0	73 ± 5.7	87 ± 3.6	95 ± 1.9	138	<0.001	***	***	***	*	***	NS
Bad drug effect	ΔE_max_	0.2 ± 0.2	6.4 ± 2.1	18 ± 3.5	14 ± 4.1	7.9	<0.001	NS	***	**	*	NS	NS
Feeling high	ΔE_max_	3.7 ± 3.7	59 ± 7.4	70 ± 7.3	77 ± 6.7	36	<0.001	***	***	***	NS	NS	NS
Fear	ΔE_max_	0.2 ± 0.1	2.3 ± 0.9	13 ± 4.1	8.2 ± 2.3	6.9	<0.001	NS	***	NS	**	NS	NS
Nausea	ΔE_max_	1.0 + 0.7	7.9 ± 2.6	25 ± 4.5	21 ± 4.8	15	<0.001	NS	***	***	***	*	NS
Alteration of vision	ΔE_max_	2.9 ± 2.8	17 ± 4.8	89 ± 4.0	87 ± 4.1	197	<0.001	*	***	***	***	***	NS
Sounds influence vision	ΔE_max_	3.2 ± 3.2	11 ± 5.2	86 ± 4.4	79 ± 6.0	119	<0.001	NS	***	***	***	***	NS
Alteration of sense of time	ΔE_max_	4.4 ± 4.2	24 ± 5.9	86 ± 5.5	92 ± 2.8	121	<0.001	**	***	***	***	***	NS
Ego dissolution	ΔE_max_	2.7 ± 2.5	15 ± 5.8	82 ± 6.2	79 ± 6.2	87	<0.001	NS	***	***	***	***	NS
Autonomic Effects													
Systolic blood pressure (mmHg)	E_max_	124 ± 2.4	148 ± 2.9	137 ± 2.7	150 ± 2.9	81	<0.001	***	***	***	***	NS	***
Diastolic blood pressure (mmHg)	E_max_	77 ± 1.7	89 ± 1.4	85 ± 1.8	90 ± 1.4	33	<0.001	***	***	***	NS	NS	*
Mean arterial pressure (mmHg)	E_max_	92 ± 1.7	107 ± 1.6	102 ± 2.0	109 ± 1.6	60	<0.001	***	***	***	**	NS	***
Heart rate (beats/min)	E_max_	77 ± 2.6	95 ± 3.4	92 ± 4.5	100 ± 3.6	25	<0.001	***	***	***	NS	NS	*
Rate pressure product (mmHg × bpm)	E_max_	9290 ± 502	13698 ± 550	12385 ± 724	14678 ± 726	43	<0.001	***	***	***	NS	NS	***
Body temperature (°C)	E_max_	37.0 ± 0.05	37.3 ± 0.07	37.4 ± 0.06	37.5 ± 0.08	27	<0.001	***	***	***	NS	*	NS
Pupil size (mm)^a^	E_max_	6.0 ± 0.2	7.1 ± 0.2	7.0 ± 0.2	7.2 ± 0.1	76	<0.001	***	***	***	NS	NS	*
Pupil size after light (mm)^a^	E_max_	4.3 ± 0.2	6.2 ± 0.2	5.7 ± 0.2	6.5 ± 0.2	4.2	<0.001	***	***	***	***	*	***
Pupil contraction (mm)^a^	E_min_	1.4 ± 0.1	0.8 ± 0.1	1.2 ± 0.1	0.6 ± 0.1	20	<0.001	***	NS	***	**	NS	***
List of Complaints (LC Score)													
Acute adverse effects	0–12 h	1.9 ± 1.1	8.6 ± 1.8	15 ± 1.9	15 ± 2.2	38	<0.001	***	***	***	***	***	NS
Subacute adverse effects	12–24 h	−0.2 ± 0.5	2.5 ± 0.9	3.1 ± 1.0	5.5 ± 1.4	11	<0.001	*	**	***	NS	*	NS
Hormones and Markers													
Oxytocin (pg/mL)	Δ1.5h	−1.6 ± 6.3	190 ± 44	30 ± 9.5	177 ± 33	14	<0.001	***	NS	***	***	NS	**
	Δ3h	−4.0 ± 5.7	212 ± 29	35 ± 8.1	246 ± 30	36	<0.001	***	NS	***	***	NS	***
	Δ6h	−12 ± 6.3	29 ± 14	36 ± 8.5	111 ± 15	22	<0.001	NS	*	***	NS	***	***
	ΔC_max_	6.0 ± 5.3	289 ± 38	60 ± 9.0	286 ± 30	45	<0.001	***	NS	***	***	NS	***
BDNF (ng/mL)	ΔC_max_	9.6 ± 1.7	7.1 ± 2.1	7.4 ± 1.7	7.4 ± 1.3	0.5	NS	–	–	–	–	–	–

*NS* not significant, *ΔE*_*max*_ maximal effect difference from baseline, *ΔE*_*min*_ minimal effect difference from baseline.

*P < 0.05; **P < 0.01; ***P < 0.001.

^a^*N* = 23.

The LSD + MDMA combination did not induce significantly different subjective responses on the VASs, 5D-ASC, MEQ, or AMRS compared with LSD alone (Figs. 1 and 2, Supplementary Figs. S1–3, Table 1, Supplementary Tables S1–4). LSD and the LSD + MDMA combination produced overall greater psychedelic effects compared with MDMA alone. LSD and the LSD + MDMA combination induced greater “any drug effects,” “good drug effects,” “ego dissolution,” “alteration of vision,” and “audio-visual synesthesia” compared with MDMA alone (Fig. 1). In contrast, ratings of “drug high” were comparable for MDMA, LSD, and the LSD + MDMA combination (Fig. 1). LSD and LSD + MDMA induced increased ratings in all main dimensions and subscales of the 5D-ASC with the exception of the subscale anxiety which only showed a trend wise increase with LSD (*p* = 0.073). MDMA only increased the subscale blissful state (Fig. 2.). LSD and the LSD + MDMA combination induced more emotional excitation, introversion, anxiety and depression compared to MDMA on the AMRS (Supplementary Fig. S3).

Subjective “any drug effects” lasted an average of 1.5 h longer after the LSD + MDMA combination (mean = 9.9 h) compared with LSD alone (mean = 8.4 h; *p* < 0.05; Fig. 1, Supplementary Table S5).

### Autonomic and adverse effects

**Fig. 3:**
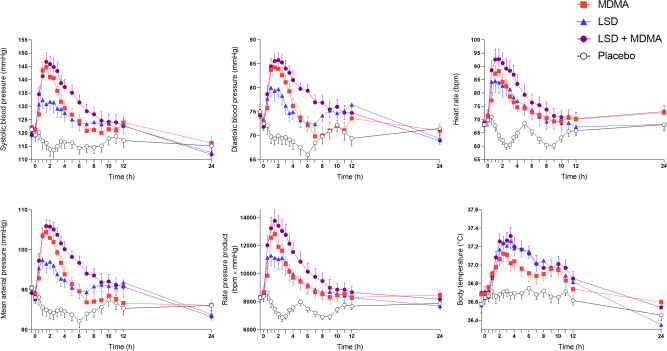
Acute autonomic effects of 100 µg lysergic acid diethylamide (LSD), 100 mg 3,4-methylenedioxymethamphetamine (MDMA), and the LSD + MDMA combination (100 µg+100 mg) over time. LSD, MDMA, and the LSD + MDMA combination increased blood pressure, heart rate, and body temperature compared with placebo. MDMA alone and the LSD + MDMA combination increased blood pressure and heart rate more compared with LSD alone. The substances were administered at t = 0 h. The data are expressed as the mean ± SEM in 24 subjects. The corresponding maximal responses and statistics are shown in Table 1.

Autonomic effects over time and respective peak effects are shown in Fig. 3 and Table 1, respectively. MDMA and the LSD + MDMA combination induced higher increases in blood pressure, heart rate, and pupil size compared with LSD alone (Fig. 3, Supplementary Fig. S4, Table 1). Body temperature increased similarly for LSD and the LSD + MDMA combination but less when MDMA was administered alone (Fig. 3). The LSD + MDMA combination and LSD alone produced similar total acute and subacute adverse effects scores on the List of Complaints, exceeding those of MDMA (Table 1). Frequently reported adverse effects on the List of Complaints are presented in Supplementary Table S6. Headache, lack of energy, loss of appetite, and dry mouth were similarly often reported with MDMA, LSD, and LSD + MDMA. Acute nausea was more frequent with MDMA than LSD. No severe adverse events were observed.

### Effects on circulating oxytocin and BDNF

Effects of MDMA, LSD, and the LSD + MDMA combination on plasma levels of oxytocin and BDNF are shown in Supplementary Fig. S5 and Table 1. MDMA alone and the LSD + MDMA combination robustly increased oxytocin, with greater peak increases compared with LSD alone. LSD alone produced only minimal increases in oxytocin. Effects of MDMA and LSD on oxytocin were additive when the two substances were combined. MDMA, LSD, and the LSD + MDMA combination had no significant effects on serum BDNF concentrations (Supplementary Fig. S5, Table 1).

### Plasma drug concentrations

The concentration-time curves for LSD, MDMA, and their metabolites are shown in Supplementary Figs. S6 and S7. Table 2 and Supplementary Table S7 show the corresponding pharmacokinetic parameters. MDMA slightly altered the pharmacokinetics of LSD. Specifically, the peak plasma concentration of LSD was higher in the LSD + MDMA condition (2.1 ng/mL) compared with LSD alone (1.9 ng/ml; T = 2.09; *p* < 0.05). The plasma LSD elimination half-life was longer in the LSD + MDMA condition (5.2 h) compared with LSD alone (3.9 h; T = 5.00; *p* < 0.001). The area under the concentration time curve (AUC_∞)_ also increased to 19 ng∙h/ml in the LSD + MDMA condition compared with LSD alone (14 ng∙h/mL; T = 3.53; *p* < 0.01; Table 2).

**Table 2:** Pharmacokinetic parameters based on non-compartmental analyses [geometric mean (95% CI), range], *N* = 24.

	C_max_ (ng/mL)	t_max_ (h)	t_1/2_ (h)	AUC_24_ (ng·h/mL)	AUC_∞_ (ng·h/mL)	CL/F (L/h)	V_z_/F (L)
LSD							
LSD + Placebo administration	1.9 (1.7–2.1)	1.6 (1.4–1.9)	3.9 (3.5–4.4)	14 (12–16)	14 (12–17)	6.9 (5.8–8.3)	39 (35–44)
	1.2–3.6	1.0–3.0	2.5–6.3	8.7–34	8.9–39	2.5–11	19–61
LSD+MDMA administration	2.1 (1.9–2.3)*	1.6 (1.4–1.9)	5.2 (4.6–5.9)***	17 (15–21)**	19 (16–22)**	5.3 (4.5–6.4)**	40 (36–45)
	1.2–3.3	1.0–3.5	2.5–8.9	8.1–33	8.3–39	2.6–12	27–77
MDMA							
MDMA + Placebo administration	233 (215–252)	2.8 (2.5–3.2)	8.3 (7.5–9.1)^a^	2596 (2333–2889)^a^	3083 (2713–3505)^a^	32 (29–37)^a^	388 (357–422)^a^
	167–306	1.5–5.0	5.0–12	1699–4143	1827–5251	19–55	274–515
LSD+MDMA administration	208 (189–228)***	3.5 (3.0–4.0)**	8.2 (7.5–9.1)	2568 (2319–2843)	3068 (2712–3472)	33 (29–37)	386 (354–422)
	130–318	2.0–6.0	5.6–16	1510–4202	1674–6540	15–60	237–574

*AUC* area under the plasma concentration-time curve, *AUC*_*∞*_ AUC from time zero to infinity, *AUC*_*24*_ from time 0 to 24, *CL/F* apparent total clearance, *C*_*max*_ maximum observed plasma concentration, *T*_*1/2*_ plasma half-life, *T*_*max*_ time to reach C_max_, *95% CI* 95% confidence interval, *V*_*z*_*/F* apparent volume of distribution.

*P < 0.05; **P < 0.01; ***P < 0.001 compared with LSD or MDMA alone, respectively (paired T-test).

^a^*N* = 23.

### Blinding

Data on the participants’ retrospective identification of their substance condition after the session and after the study are shown in Supplementary Table S8. During and after receiving LSD + MDMA, 50% and 46% of the participants, respectively, thought they received LSD alone. During and after receiving LSD alone, 25% and 38% of the participants, respectively, thought they received LSD + MDMA. When asked at the end of the study, 25% of the participants mistook LSD for LSD + MDMA and vice versa.

## Discussion

The main finding of the present study was that MDMA co-administration did not relevantly alter acute psychedelic effects of LSD while producing greater autonomic effects compared with LSD alone. However, LSD + MDMA co-administration prolonged acute subjective effects compared with LSD alone. The prolonged LSD response is consistent with a higher plasma concentration of LSD (C_max_ and AUC) and a longer plasma elimination half-life of LSD when it was co-administered with MDMA and as determined in the present study. Acute effects of LSD and MDMA alone have previously been compared in healthy participants [3], but the present study was the first to investigate the combined use of MDMA and LSD in a controlled laboratory setting and using defined doses of both substances. Synergistic discriminative effects of LSD and MDMA were previously reported in rats [24]. However, the rats were trained to discriminate MDMA (1.5 mg/kg) alone from saline, and then the co-administration of a low MDMA dose (0.15 mg/kg) with LSD (0.04 mg/kg) produced a full MDMA-like response [24]. Acute subjective effects of LSD are primarily positive. However, there are also negative subjective effects (e.g., anxiety) of LSD, depending on the dose of LSD used, personality traits of the person using LSD, their life circumstances, and the setting [1, 3–7, 9, 36]. Acute negative psychological effects are the main adverse events that are associated with LSD when it is used in psychedelic-assisted therapy [1]. In contrast to LSD, MDMA induces fewer psychedelic effects with little anxious ego-dissolution [3]. MDMA typically produces robust positive subjective effects, including enhanced feelings of positive mood, well-being, empathy, trust, and closeness to others [3, 16–19].

Therefore, we hypothesized that adding MDMA to LSD would enhance positive mood effects and decrease anxiety that is associated with the LSD response. The same approach is also used by recreational substance users when combining MDMA and LSD in “candyflipping.” Contrary to our expectation, the present controlled study showed that the co-administration of LSD and MDMA and administration of LSD alone produced overall very similar subjective effects on the VAS, 5D-ASC, and MEQ. However, although no significant differences were seen, the addition of MDMA tended to nonsignificantly increase ratings of “happy,” “open,” and “trust” on the VAS and “well-being” on the AMRS, especially in the beginning of the experience compared with LSD alone. Additionally, ratings of “well-being” on the AMRS increased at the beginning of the drug response but dropped at 6 h when the MDMA effect ended. This may indicate some enhanced MDMA-typical subjective effects with the combination compared with LSD alone. Furthermore, we only tested single dose levels of both LSD and MDMA and co-administration at the same time. An LSD base dose of 100 µg has previously been used in several studies in healthy participants [3, 8, 36, 37] and could be considered a moderately high dose. LSD at a dose of 100 µg mainly induces high acute positive effects and nominally less anxiety compared with a higher dose of 200 µg [7, 36]. Thus, we cannot exclude the possibility that MDMA may reduce negative mood effects, including anxiety, of higher LSD doses than the dose that was used in the present study. The MDMA dose of 100 mg was lower than the 120–125 mg doses that were mostly used in healthy research participants [19] and patients [16]. A 100 mg dose of MDMA that is administered in women is equivalent to 120–125 mg in men and can be considered a fully psychoactive dose in women when given alone [19, 38] and not co-administered with LSD. Nevertheless, we cannot exclude different interactive effects of MDMA and LSD at different dose levels and administration time-points than those that were used herein. The duration of the acute LSD response is longer than the MDMA response, as confirmed in the present study. Future studies may test the administration of MDMA 1–4 h after LSD or use a prolonged MDMA release formulation or pro-drug of MDMA to better align its effects with the time course of the LSD effect. Moreover, the combination of MDMA and psilocybin may be interesting because of their similar durations of action [3, 36]. However, average peak effects of MDMA and LSD were reached at similar times in the present study, indicating a good match of the two subjective effect-time curves over the first 4 h. The potential drop in positive MDMA effects might have resulted in more negative mood states from 5 to 12 h in some participants, indicated by the trend-wise lower “well-being” ratings on the AMRS and higher “depression” ratings on the AMRS toward the end of the LSD response when it was co-administered with MDMA. Notably, recreational users reportedly often take MDMA after LSD when “candyflipping.”

LSD, MDMA, and their combination produced significant autonomic stimulant effects as reported previously [3, 9, 39]. The LSD + MDMA combination induced greater increases in blood pressure and heart rate compared with LSD alone. Body temperature increased similarly after LSD + MDMA co-administration and LSD administration alone and more after LSD + MDMA co-administration compared with MDMA administration alone.

MDMA had no relevant effects on the quality of the acute response to LSD, whereas the LSD + MDMA combination resulted in a longer effect duration compared with LSD and MDMA alone. This can be explained by higher plasma concentrations (both C_max_ and AUC) and a longer plasma elimination half-life of LSD when it was co-administered with MDMA. Thus, MDMA and LSD primarily interact pharmacokinetically and not pharmacodynamically. Additionally, the higher plasma exposure to LSD could be explained by metabolic P450 enzyme CYP2D6 inhibition by MDMA [38, 40]. MDMA is a strong inhibitor of CYP2D6, turning any CYP2D6 extensive or rapid metabolizer into a poor metabolizer within approximately 2 h [41]. Additionally, CYP2D6 poor metabolizers exhibited higher plasma concentrations and a longer elimination half-life of LSD compared with extensive metabolizers [42]. Thus, the present study further confirms a role for CYP2D6 in the metabolism of LSD. A similar or substantial increase in plasma LSD concentrations could be expected when patients who are on antidepressants that inhibit CYP2D6 (e.g., fluoxetine, paroxetine, duloxetine, and bupropion) and are treated with LSD-assisted therapy. This interaction warrants further study.

We also evaluated selected interactive endocrine effects of LSD and MDMA. The marked release of oxytocin may mediate some of subjective effects of MDMA [17, 43, 44]. LSD also increased circulating oxytocin, although not robustly and to a lower extent than MDMA [3, 6, 7]. In the present study, effects of MDMA and LSD on plasma oxytocin concentrations were additive. Neither MDMA nor LSD altered serum concentrations of BDNF, adding further data to several inconclusive studies [3, 7, 8, 45].

The present study also provided insights into the ways in which neurotransmitters mediate subjective effects of psychoactive substances. LSD directly activates the serotonin 5-hydroxytryptamine-2A (5-HT_2A_) receptor [46], which primarily mediates its acute psychedelic effects [7, 8, 47]. MDMA induces the release of endogenous norepinephrine, serotonin, and oxytocin [44, 48, 49]. The present study indicates that stimulating serotonin and norepinephrine with the empathogen MDMA, in addition to the direct activation of 5-HT_2A_ receptors by the psychedelic LSD, does not relevantly alter the subjective effects profile of a psychedelic alone. This finding is also consistent with the observation that LSD alone strongly exerts several MDMA-like empathogenic effects, including similar ratings of well-being, happiness, closeness to others, openness, and trust, as previously reported [3, 6] and confirmed in the present study. Interestingly, the additional release of serotonin and oxytocin by MDMA does not appear to result in relevant additional psychoactive effects of LSD. The additional release of norepinephrine by MDMA explains the greater cardiovascular stimulation after the co-administration of LSD and MDMA compared with LSD alone.

We found no indication of greater serotonin toxicity when MDMA and LSD were co-administered. MDMA did not increase thermogenic effects of LSD alone. Nausea was similarly frequent after the co-administration of LSD and MDMA and the administration of either substance alone.

The role of dopamine in subjective effects of LSD remains unclear [50, 51]. LSD binds to dopamine D_1_ and D_2_ receptors [46]. We consider direct dopamine receptor stimulation irrelevant for psychedelic properties of LSD because subjective effects of LSD can be fully antagonized by blocking 5-HT_2_ receptors [7, 8] and are very comparable to subjective effects of psilocybin [36] (a psychedelic with no relevant effects on D_1_ or D_2_ receptors) [46]. MDMA also induces the release of dopamine [52], and this may explain the nominally greater well-being ratings after the co-administration of MDMA and LSD compared with LSD alone.

The present study has several strengths. We used a relatively large study sample (*n* = 24) and powerful within-subject comparisons in a randomized double-blind design. The LSD and MDMA doses were pharmacologically well characterized. We included equal numbers of male and female participants and used internationally established psychometric outcome measures. Plasma LSD and MDMA concentrations were determined at close intervals in all participants and analyzed with validated analytical methods.

Notwithstanding these strengths, the present study also has limitations. We used only one dose of LSD and MDMA. The study used a highly controlled hospital setting and included only healthy volunteers. Thus, people in different environments and patients with psychiatric disorders may respond differently to these substances. Finally, the outcome measures may not have been sufficiently sensitive to capture all aspects of a psychedelic experience and/or very subtle differences between acute effects of LSD + MDMA compared with LSD alone.

## Conclusion

MDMA co-administration did not alter acute psychedelic effects of LSD. However, MDMA acted as a blocker of the metabolism of LSD to prolong its presence in the body and acute effects. The LSD + MDMA combination produced more autonomic effects compared with LSD alone. There is likely little benefit in combining MDMA and LSD in psychedelic-assisted therapy.

### Supplementary information

Supplement

CONSORT Flowchart
