# Supplementary material for: Acute effects of MDMA and LSD co-administration in a double-blind placebo-controlled study in healthy participants
Source: Neuropsychopharmacology. 2023 May 31;48(13):1840–8. doi: 10.1038/s41386-023-01609-0 (PMC10584820; doi:10.1038/s41386-023-01609-0)
Supplement: Supplementary file 1 — Supplement [file 41386_2023_1609_MOESM1_ESM.pdf]

Supplement

Methods

Subjective drug effects measurements
Visual Analog Scales (VASs)

Subjective effects were assessed repeatedly using visual analog scales (VASs) [1,2]. 0.5 h before and 0, 0.5, 1, 1.5, 2, 2.5, 3, 3.5, 4, 5, 6, 7, 8, 9, 10, 11, 12 and 24 h after drug administration. The VASs included “any drug effect”, “good drug effect”, “bad drug effect”, “feeling high”, “fear”, “nausea”, “alteration of vision”, “sounds seem to influence what I see”, “alteration of sense of time” and “ego dissolution,” that were presented as 100-mm horizontal lines (0-100%), marked from “not at all” on the left to “extremely” on the right [1,3]. Further VASs included “feeling emotional”, “happy”, “talkative”, “open”, “trust”, “I feel close to others” “I want to be alone”, “I want to be with others” and “my focus is directed inward/outward”. These VASs were bidirectional and marked with “normal” in the middle at 0 mm and “not at all” (-50 mm) on the left and “extremely” (50 mm) on the right. The primary VAS outcome measures were “good drug effect”, “trust”, “open”, “bad drug effects” and “fear”. These VASs have been repeatedly used and shown to be sensitive with MDMA and LSD [1-6]. The VAS can be completed relatively rapidly and easily by the participant even during the LSD/MDMA experience and allows for a valid prospective definition of the drug effects over time. They are sensitive and relatively simple measures. More complex assessments of the state of LSD and MDMA have to be performed primarily at the end of the session and include entire multi-item questionnaires. The VAS “any drug effect” is an overall effect measure to characterize the overall effect intensity and time course. The VAS “good drug effect” is an overall measure of effects subjectively considered positive and interrelated with other measures such as “drug liking”. The VAS “bad drug effect” is an overall measure of any negative effects and related to “fear”. Typically, “bad drug effects” of LSD tend to occur only at higher doses or plasma concentrations according to previous PK-PD analyses [4,5]. The VAS “ego dissolution” was marked with the sentence: “the boundaries between myself and my surroundings seemed to blur”. This is also an item of the 5D-ASC (no. 71) which has been used as a simple measure of “ego dissolution” previously [7,8] and can be used repeatedly as a single VAS [1,4]. VASs were assessed each time LSD and MDMA blood concentrations were measured.

Adjective Mood Rating Scale (AMRS)

The Adjective Mood Rating Scale (AMRS) [9] was used 0.5 h before and 3, 6, 9, 12, and 24 h after drug administration. The AMRS is a validated 60-item Likert mood rating scale mainly use in Europe and consists of subscales including ratings on “well-being”, “anxiety”, “inactivity”, “extraversion”, “introversion”, and “emotional excitation”. It is suitable for repeated measurements of mood states. The short German EWL60S version was used [9]. The completion of the ratings under the effects of psychedelics substances is possible but difficult because it lasts several minutes. The scale was used in paper and pencil version but it may be more suitable to use this measure verbally during states of markedly impaired concentration. The AMRS was included as a primary measure because it could be considered a better validated measure of mood states and producing more defined ratings than the VAS and to support findings on the VAS (AMRS well-being considered similar to VAS good drug effects; AMRS anxiety considered similar to VAS fear).

5 Dimension of Altered States of Consciousness (5D-ASC) scale

The 5 Dimensions of Altered States of Consciousness (5D-ASC) scale [10,11] was used as the primary outcome measure and was administered 12 h after drug administration to retrospectively rate peak drug effects. The 5D-ASC scale measures altered states of consciousness and contains 94 items (visual analog scales). The instrument consists of five subscales/dimensions [10] and 11 lower-order scales [11]. The 5D-ASC dimension “Oceanic

Boundlessness” (27 items) measures derealization and depersonalization associated with positive emotional states, ranging from heightened mood to euphoric exaltation. The corresponding lower-order scales include “experience of unity,” “spiritual experience,” “blissful state,” “insightfulness,” and “disembodiment.” The dimension “Anxious Ego Dissolution” (21 items) summarizes ego-disintegration and loss of self-control phenomena associated with anxiety. The corresponding lower-order scales include “impaired control of cognition” and “anxiety.” The dimension “Visionary Restructuralization” (18 items) consists of the lower-order scales “complex imagery,” “elementary imagery,” “audio-visual synesthesia,” and “changed meaning of percepts.” Two additional dimensions describe “Auditory Alterations” (15 items) and “Reduction of Vigilance” (12 items). The total 3D-ASC score is the total of the three main dimensions “Oceanic Boundlessness”, “Anxious Ego-Dissolution”, and “Visionary Restructuralization” and can be used as a measure of the overall intensity of the alteration of the mind [8]. The scale is well-validated in German [10] and many other languages and widely used to characterize the subjective effects of various psychedelic drugs. In particular, the scale has been used by most research groups to psychometrically assess LSD and MDMA effects [1,2,12-16]. Furthermore, acute ratings on the 5D-ASC after administration of psilocybin have been used to predict long-term effects of psychedelic treatments in patients [17,18]. Ratings on the 5D-ASC have been shown to closely correlate with ratings on the Mystical Effects Questionnaire (MEQ, see below) [8] which is primarily used by research groups in the US [18].

Mystical Effects Questionnaire (MEQ30)

Mystical experiences were assessed 12 h after drug administration using the 100-item States of Consciousness Questionnaire (SOCQ) [8,19] that includes the 43-item Mystical Effects Questionnaire (MEQ43) [19], 30-item Mystical Effects Questionnaire (MEQ30) [20], and subscales for “aesthetic experience” and negative “nadir” effects. The published German version was used [8]. The MEQ has been used in numerous experimental and therapeutic trials with psilocybin [18,19,21-27]. The MEQ items provide scale scores for each of seven domains of mystical experiences: internal unity, external unity, sacredness, noetic quality (as real as or more real than everyday reality), deeply felt positive mood, transcendence of time and space, and ineffability/paradoxicality (difficulty describing the experience in words). The total of all scale scores was used as an overall measure of the mystical-type experience. We also derived the four scale scores of the newly validated revised 30-item MEQ: mystical, positive mood, transcendence of time and space, and ineffability [20]. A complete mystical experience was defined as scores ≥ 60% on all MEQ30 factors [20]. While we prefer the German 5D-ASC scale, the German version of the MEQ was also included to facilitate comparison of our findings with those from research using the MEQ (mainly US). Additionally, some aspects of the LSD experience may be better captured with this scale. For the scale validation see [20]. For an analysis of the interrelation of the two measures with regards to responses to LSD see [8]. For the German translation of the MEQ30 see online supplement of [8].

Brain-derived neurotropic factor measurements

Serum concentrations of brain-derived neurotrophic factor (BDNF) were measured using an enzyme-linked immunosorbent assay (ELISA) kit (Biosensis Mature BDNF Rapid ELISA Kit: Human, Mouse, Rat; Thebarton, SA, Australia). Blood samples were drawn using serum tubes. After 30 min at room temperature, the serum tubes were centrifuged at 14,100 × *g* for 10 min at 4°C. Samples were stored at -80°C before the analysis of BDNF levels. Serum samples were diluted (1:100), and BDNF was detected on a pre-coated mouse monoclonal anti-mature BDNF 96-well plate as described in the manufacturer’s protocol. Absorbance was read at 450 nm within 5 min after adding the stop solution in a microplate reader. The correction wavelength was set to 690 nm to determine BDNF concentrations according to the standard curve that was calculated from a 4-parameter logistics curve fit.

Results

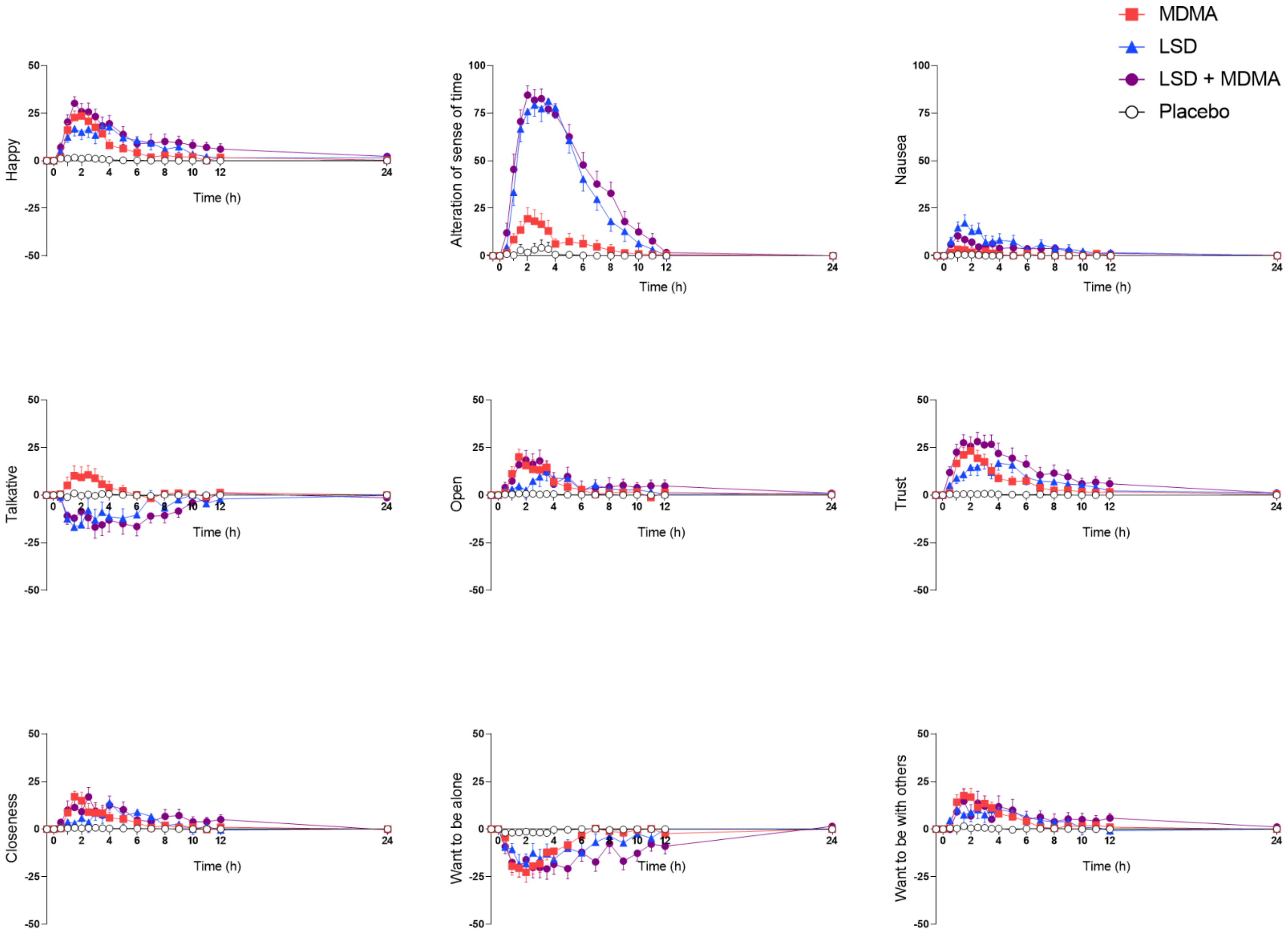
MDMA
LSD
LSD + MDMA
Placebo
Happy
Alteration of sense of time
Nausea
Talkative
Open
Trust
Closeness
Want to be alone
Want to be with others
Time (h)

Figure S1. Acute subjective effects induced by 3,4-Methylendioxymethamphetamin (MDMA), lysergic acid diethylamide (LSD) and the combination of LSD and MDMA over time on the Visual Analog Scale (VAS). MDMA (100 mg), LSD (100 µg), and LSD & MDMA (100 µg, 100 mg), or placebo was administered at t = 0 h. Generally, LSD and the co-administration of LSD and MDMA produced comparable subjective effects on all depicted VASs. MDMA alone also showed comparable subjective effects except on the VAS “alteration of sense of time” and “talkative”. The data are expressed as the mean ± SEM percentage of maximally possible scale scores in 24 subjects. The corresponding maximal responses and statistics are shown in Table 1 and in Supplementary Table S1.

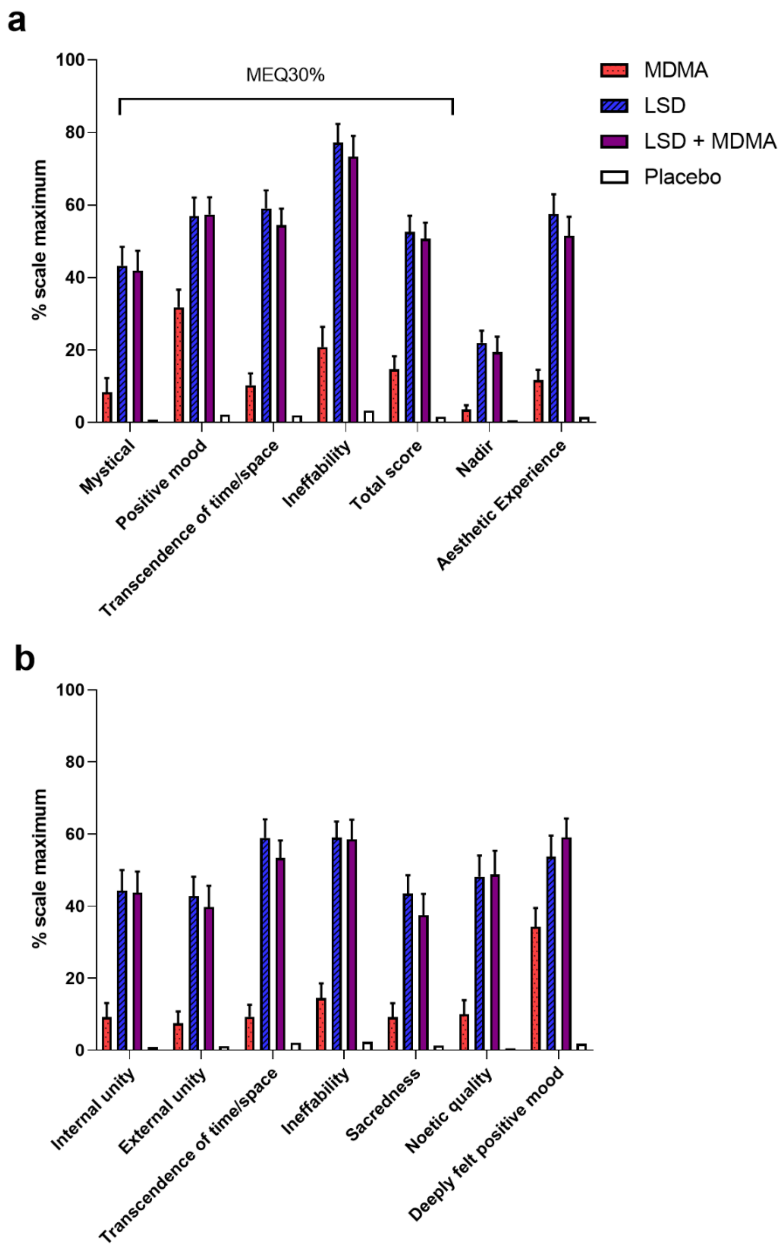

Figure S2. Acute mystical-type experiences on the Mystical Effects Questionnaire (MEQ). The combination of 100 µg LSD with 100 mg MDMA induced overall comparable effects to 100 µg of LSD alone on the MEQ30 (**a**) and the MEQ43 (**b**). 100 mg MDMA alone only showed significant mystical-type effects for the positive mood subscales on both the MEQ30 and MEQ45. Ineffability in the MEQ30 and the MEQ30 total score were significant as well for MDMA alone. The data are expressed as the mean ± SEM percentage of maximally possible scale scores in 24 subjects. Statistics are shown in Supplementary Table S3.

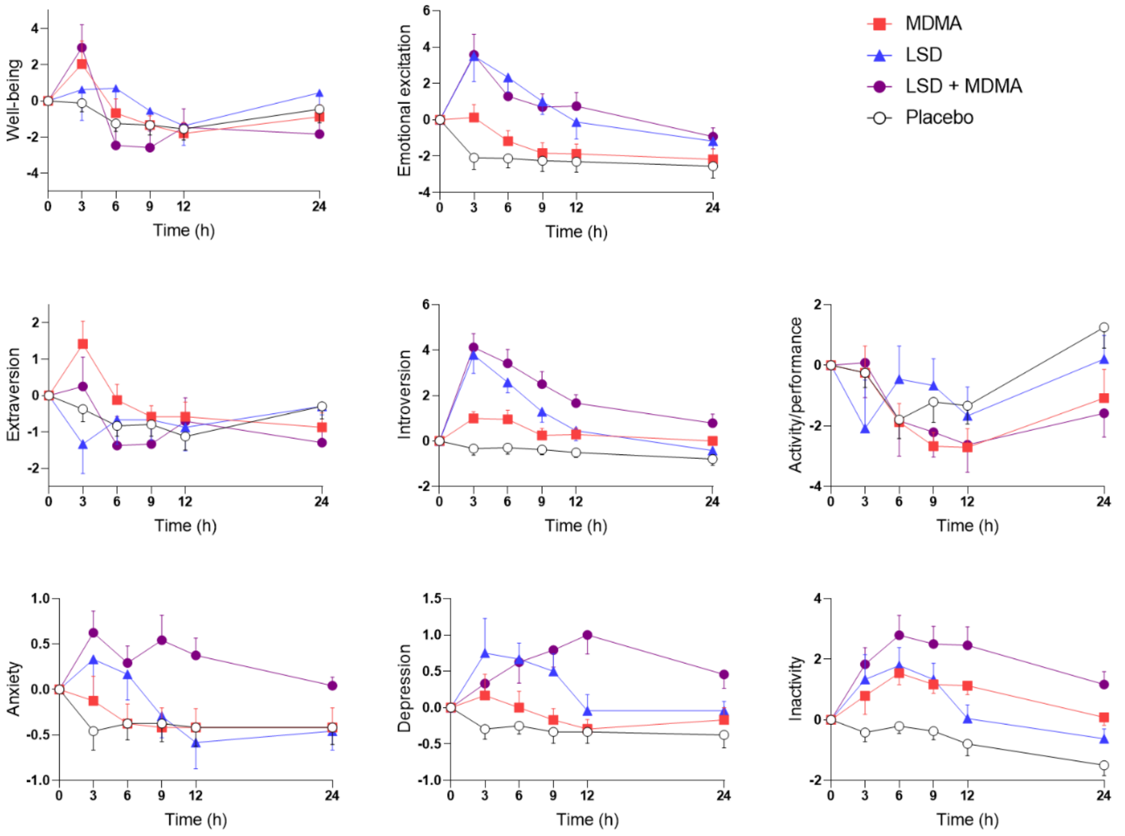

Figure S3. Subjective effects over time on the Adjective Mood Rating Scale (AMRS). The data are expressed as mean ± SEM changes from baseline. All conditions nominally reduced activity ratings on the AMRS with no difference between placebo and either active drug condition. All substance conditions significantly increased inactivity ratings compared with placebo. LSD and LSD with co-administered MDMA significantly altered peak well-being ratings compared with placebo. While all substance conditions increased introversion compared with Placebo, LSD and the combination of LSD and MDMA increased introversion compared to MDMA alone as well. Emotional excitation was enhanced with LSD and the co-administered LSD and MDMA compared to Placebo and MDMA alone. LSD as well as LSD with MDMA combined both significantly increased self-rated anxiety compared to Placebo and MDMA alone. The combined LSD and MDMA and LSD alone increased self-rating of depression compared to Placebo and MDMA alone. LSD (100 µg), LSD & MDMA (100 µg, 100 mg), MDMA (100 mg) or placebo was administered at t = 0 h. The data are expressed as the mean ± SEM percentage of maximally possible scale scores in 24 subjects. The corresponding maximal effects and statistics are shown in Supplementary Table S4.

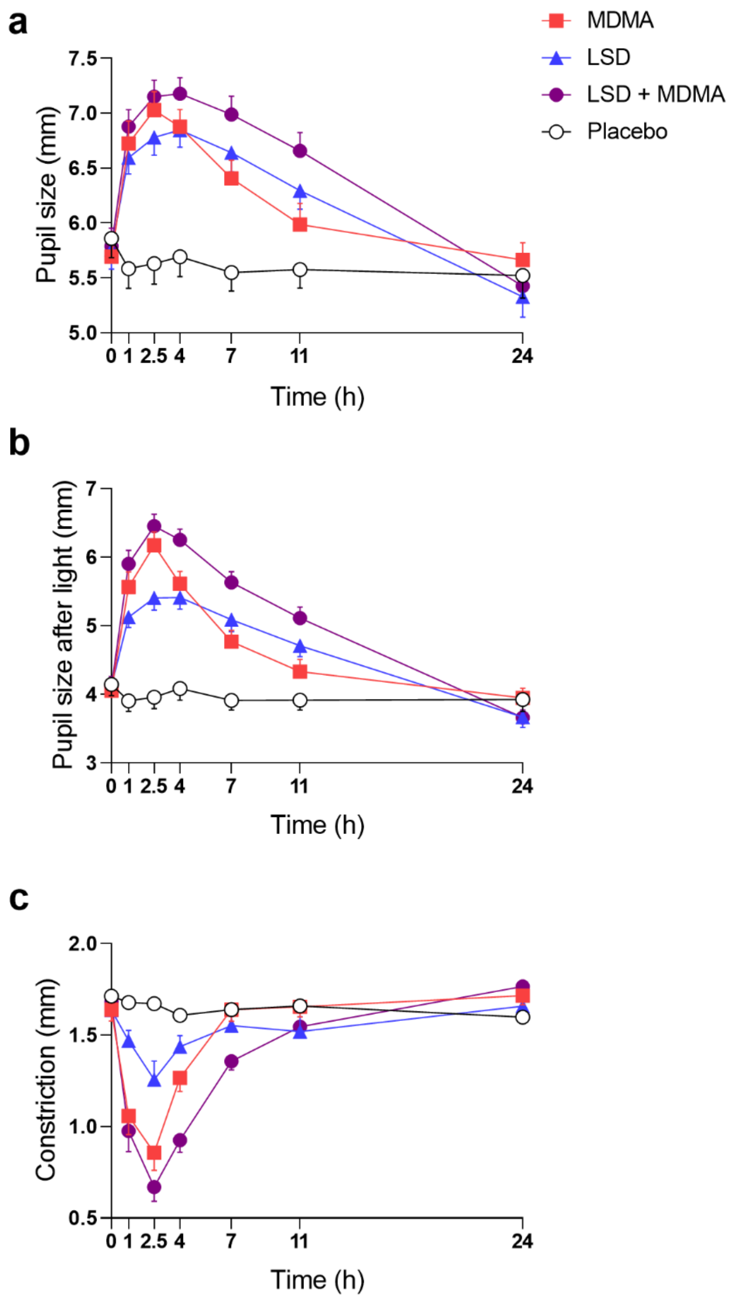

Figure S4. Effects of lysergic acid diethylamide (LSD), 3,4-Methylendioxymethamphetamin (MDMA), and the combination of LSD and MDMA over time on pupillary function. All conditions increased pupil size compared to placebo, additionally (**a-b**) at all doses and reduced the reaction to light (**c**). The reduction of the pupillary constriction in response to light was statistically significant more pronounced with LSD & MDMA and MDMA alone compared to LSD. LSD (100 µg), MDMA (100 mg), LSD & MDMA (100 µg, 100 mg) or placebo was administered at t = 0 h. The data are expressed as the mean ± SEM in 23 subjects. The corresponding maximal effects and statistics are shown in Table 1.

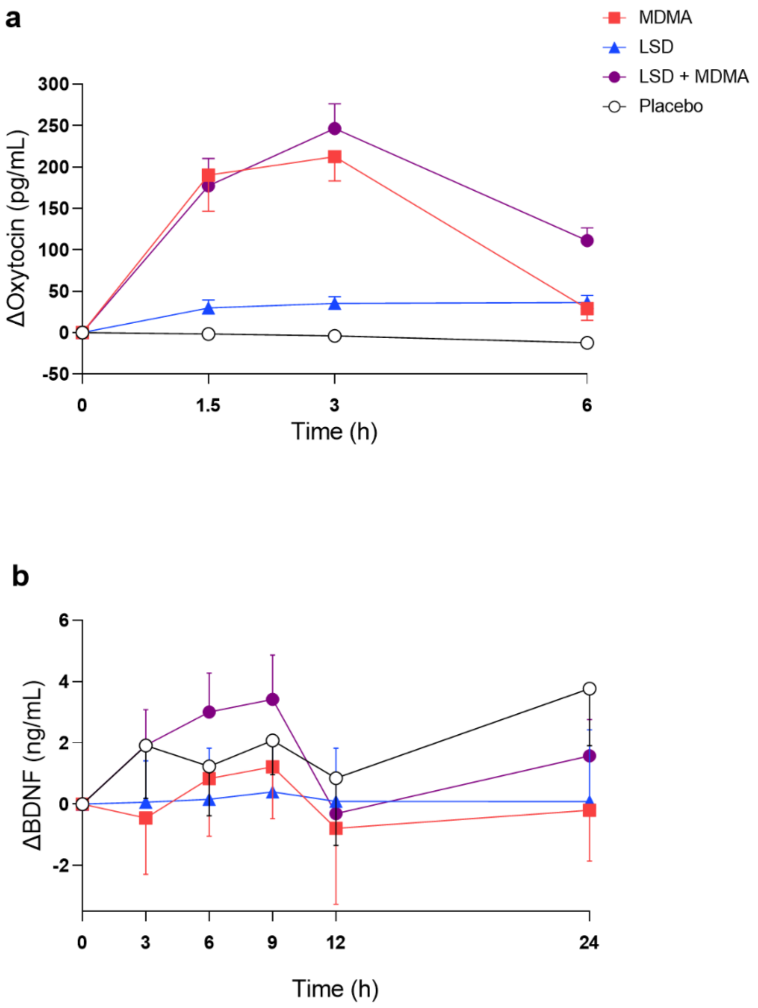

Figure S5. Plasma concentrations of oxytocin (**a**), and serum concentrations of Brain-Derived Neurotrophic Factor (**b**, BDNF). The data are expressed as mean ± SEM. LSD (100 µg), MDMA (100 mg), LSD & MDMA (100 µg, 100 mg) or placebo was administered at t = 0 h. The corresponding maximal effects and statistics are shown in Table 1.

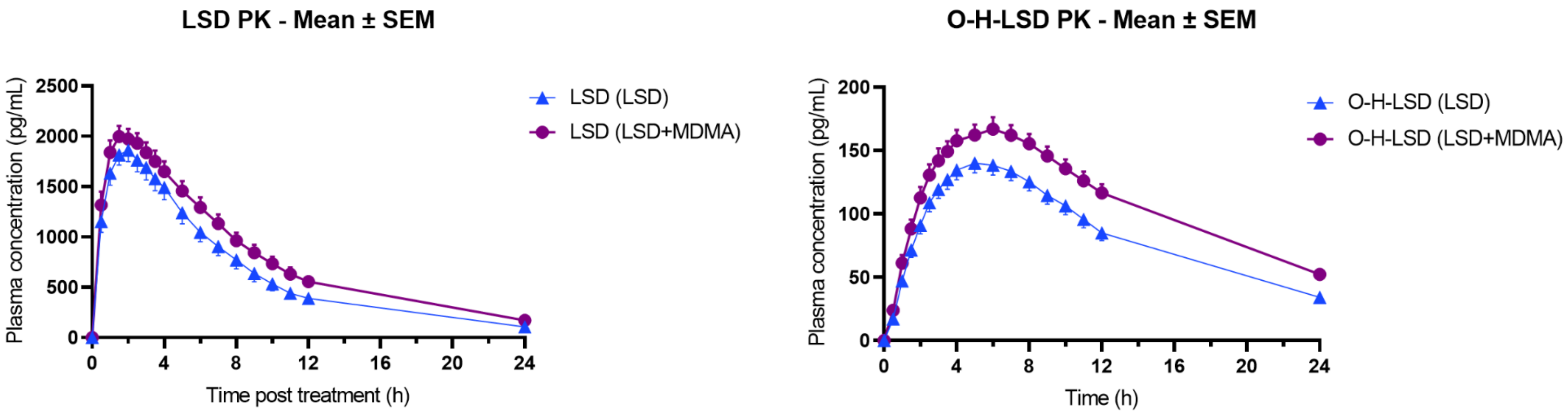

Figure S6. Plasma concentrations of LSD and its main metabolite 2-oxo-3-hydroxy-LSD (O-H-LSD) with and without MDMA. The data are expressed as mean ± SEM. LSD (100 µg) or LSD & MDMA (100 µg, 100 mg) was administered at t = 0 h. The corresponding pharmacokinetic parameters were determined by non-compartmental analysis and are shown in Table 2 and Table S7.

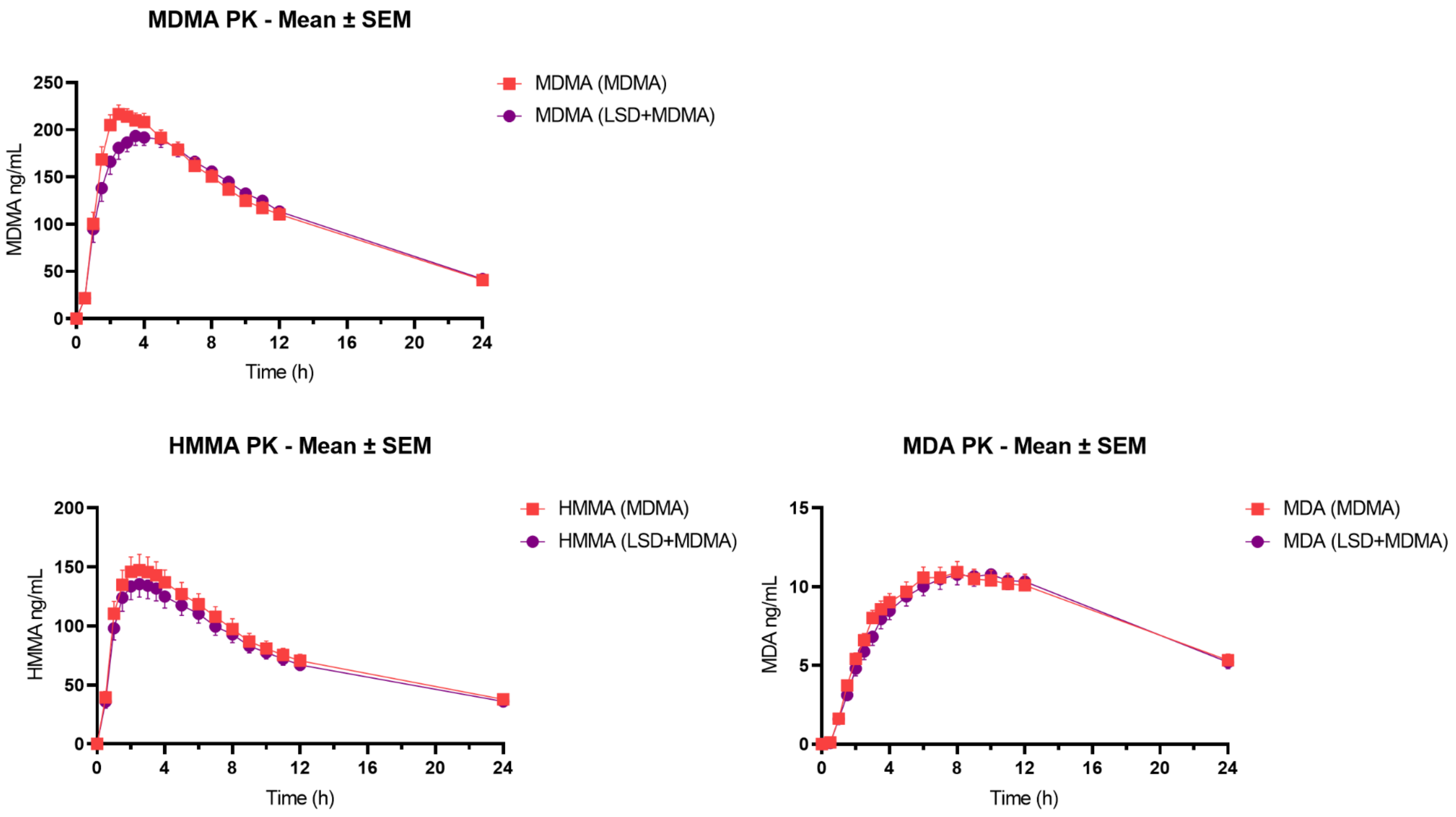

Figure S7. Plasma concentrations of MDMA and the metabolites 4-Hydroxy-3-methoxymethamphetamine (HMMA) and 3,4-methylenedioxyamphetamine (MDA) with and without LSD. HMMA concentrations were determined after enzymatic deglucuronidation. The data are expressed as mean ± SEM. MDMA (100 mg) or LSD & MDMA (100 µg, 100 mg) was administered at t = 0 h. The corresponding pharmacokinetic parameters were determined by non-compartmental analysis and are shown in Table 2 and Table S7.

Table S1. Mean values and statistics for the acute subjective effects on the Visual Analog Scale (VAS)

		Placebo mean±SEM	MDMA mean±SEM	LSD mean±SEM	LSD+MDMA mean±SEM	$F_{3, 69}$	P=	Pla - MDMA	Pla - LSD	Pla - MDMA + LSD	MDMA - LSD	MDMA - MDMA + LSD	LSD - MDMA + LSD
Visual Analog Scale (VAS, %max)													
Bidirectional Scales (-50 to 50)													
Emotional	ΔE_{max}	1.5 ± 1.2	21 ± 3.4	28 ± 3.9	27 ± 4.0	22	<0.001	***	***	***	NS	NS	NS
	ΔE_{min}	0 ± 0	-2.3 ± 1.1	-5.3 ± 2.7	-5.3 ± 2.7	1.7	NS	-	-	-	-	-	-
Happy	ΔE_{min}	2.0 ± 1.3	31 ± 3.3	31 ± 3.8	38 ± 3.2	37	<0.001	***	***	***	NS	NS	NS
	ΔE_{min}	0 ± 0	-2.5 ± 1.4	-5.9 ± 2.0	-6.5 ± 2.5	3.2	0.029	NS	NS	*	NS	NS	NS
Talkative	ΔE_{max}	1.3 ± 1.3	24 ± 4.1	16 ± 3.5	22 ± 3.9	13	<0.001	***	**	***	NS	NS	NS
	ΔE_{min}	-1.4 ± 0.8	-14 ± 2.8	-40 ± 2.7	-40 ± 2.7	88	<0.001	***	***	***	***	***	NS
Open	ΔE_{max}	0.9 ± 0.9	27 ± 3.6	26 ± 3.7	32 ± 3.7	28	<0.001	***	***	***	NS	NS	NS
	ΔE_{min}	0 ± 0	-8.0 ± 2.8	-15 ± 3.6	-19 ± 4.1	9.3	<0.001	NS	**	***	NS	*	NS
Trust	ΔE_{max}	0.8 ± 0.8	29 ± 3.6	31 ± 3.8	38 ± 3.1	46	<0.001	***	***	***	NS	NS	NS
	ΔE_{min}	0 ± 0	-1.1 ± 1.0	-6.1 ± 2.7	-5.6 ± 2.8	3.2	NS	-	-	-	-	-	-
I feel close to others	ΔE_{max}	0.8 ± 0.8	25 ± 3.4	24 ± 3.9	29 ± 3.5	27	<0.001	***	***	***	NS	NS	NS
	ΔE_{min}	0 ± 0	-8.2 ± 3.1	-14 ± 3.2	-16 ± 3.4	7.4	<0.001	NS	**	***	NS	NS	NS
I want to be alone	ΔE_{max}	0.2 ± 0.2	4.9 ± 1.7	10 ± 2.5	13 ± 3.2	8.0	<0.001	NS	**	***	NS	*	NS
	ΔE_{min}	-2.1 ± 2.1	-33 ± 4.0	-36 ± 3.8	-36 ± 4.0	32	<0.001	***	***	***	NS	NS	NS
I want to be with others	ΔE_{max}	1.5 ± 1.5	28 ± 4.0	21 ± 4.1	30 ± 4.3	21	<0.001	***	***	***	NS	NS	NS
	ΔE_{min}	-0.3 ± 0.3	-3.8 ±1.5	-16 ± 3.5	-20 ± 4.2	13	<0.001	NS	***	***	**	***	NS

*P<0.05, **P<0.01, ***P<0.001; NS, not significant; ΔE_{max}, maximal effect difference from baseline; ΔE_{min}, minimal effect difference from baseline; N=24

Table S2. Mean values and statistics for the acute subjective effects on the 5 Dimensions of Altered States of Consciousness (5D-ASC) Scale

		Placebo mean±SEM	MDMA mean±SEM	LSD mean±SEM	LSD+MDMA mean±SEM	$F_{3,69}$	P=	Pla - MDMA	Pla - LSD	Pla - MDMA + LSD	MDMA - LSD	MDMA - MDMA + LSD	LSD - MDMA + LSD
5 Dimensions of Altered States of Consciousness (5D-ASC) Scale													
5D-ASC total Score	% score	1.1 ± 1.1	7.3 ± 2.3	34 ± 3.4	37 ± 3.9	69	<0.001	NS	***	***	***	***	NS
3D-ASC total Score	% score	1.3 ± 1.3	7.8 ± 2.7	38 ± 4.0	41 ± 4.5	65	<0.001	NS	***	***	***	***	NS
Oceanic boundlessness	% score	1.5 ± 1.5	13 ± 4.1	46 ± 5.4	53 ± 5.3	49	<0.001	NS	***	***	***	***	NS
Anxious ego-dissolution	% score	0.6 ± 0.6	1.8 ± 0.6	20 ± 3.1	22 ± 4.4	21	<0.001	NS	***	***	***	***	NS
Visionary restructuralization	% score	1.6 ± 1.6	6.4 ± 3.4	48 ± 5.0	47 ± 5.9	58	<0.001	NS	***	***	***	***	NS
Auditory alterations	% score	0.4 ± 0.4	0.9 ± 0.6	11 ± 3.3	13 ± 3.6	8.0	<0.001	NS	*	**	*	**	NS
Reductions of vigilance	% score	1.5 ± 1.5	14 ± 3.6	43 ± 5.0	46 ± 5.8	43	<0.001	NS	***	***	***	***	NS
Experience of unity	% score	1.0 ± 1.0	7.0 ± 3.8	48 ± 6.1	51 ± 6.8	37	<0.001	NS	***	***	***	***	NS
Spiritual experience	% score	0 ± 0	5.3 ± 4.2	27 ± 5.9	32 ± 6.4	14	<0.001	NS	***	***	**	***	NS
Blissful state	% score	2.9 ± 2.9	33 ± 7.0	50 ± 7.9	68 ± 6.2	23	<0.001	**	***	***	NS	***	NS
Insightfulness	% score	0 ± 0	12 ± 4.8	39 ± 6.8	45 ± 7.2	21	<0.001	NS	***	***	***	***	NS
Disembodiment	% score	0.5 ± 0.5	5.6 ± 3.8	39 ± 6.2	40 ± 7.9	19	<0.001	NS	***	***	***	***	NS
Impaired control and cognition	% score	1.3 ± 1.3	3.6 ± 1.3	30 ± 4.4	35 ± 5.6	29	<0.001	NS	***	***	***	***	NS
Anxiety	% score	0.2 ± 0.2	0.3 ± 0.2	9.9 ± 3.4	9.3 ± 4.4	3.8	0.014	NS	NS	NS	NS	NS	NS
Complex imagery	% score	0.8 ± 0.8	6.5 ± 3.5	56 ± 7.5	52 ± 8.2	34	<0.001	NS	***	***	***	***	NS
Elementary imagery	% score	1.5 ± 1.5	2.7 ± 2.5	59 ± 7.1	61 ± 8.8	43	<0.001	NS	***	***	***	***	NS
Audio-visual synesthesia	% score	1.9 ± 1.9	4.2 ± 3.4	62 ± 6.7	62 ± 8.2	48	<0.001	NS	***	***	***	***	NS
Changed meaning of percepts	% score	2.9 ± 2.9	6.7 ± 3.7	34 ± 5.9	38 ± 6.2	25	<0.001	NS	***	***	***	***	NS

*P<0.05, **P<0.01, ***P<0.001; NS, not significant; N=24

Table S3. Mean values and statistics for the acute subjective effects on the Mystical Experience Questionnaire (MEQ)

		Placebo mean±SEM	MDMA mean±SEM	LSD mean±SEM	LSD+MDMA mean±SEM	$F_{3,69}$	P=	Pla - MDMA	Pla - LSD	Pla - MDMA + LSD	MDMA - LSD	MDMA - MDMA + LSD	LSD - MDMA + LSD
Mystical Experiences Questionnaire (MEQ30)													
Mystical	% score	0.8 ± 0.8	8.3 ± 3.9	43 ± 5.2	42 ± 5.6	34	<0.001	NS	***	***	***	***	NS
Positive mood	% score	2.2 ± 2.2	32 ± 4.9	57 ± 5.1	57 ± 4.8	47	<0.001	***	***	***	***	***	NS
Transcendence of time/space	% score	1.9 ± 1.9	10 ± 3.3	59 ± 5.0	54 ± 4.6	87	<0.001	NS	***	***	***	***	NS
Ineffability	% score	3.3 ± 3.3	21 ± 5.5	77 ± 5.1	73 ± 5.7	76	<0.001	*	***	***	***	***	NS
MEQ30 total score	% score	1.6 ± 1.6	15 ± 3.6	53 ± 4.5	51 ± 4.5	66	<0.001	*	***	***	***	***	NS
Nadir effects	% score	0.6 ± 0.6	3.6 ± 1.2	22 ± 3.3	19 ± 4.3	20	<0.001	NS	***	***	***	***	NS
Aesthetic Experience	% score	1.5 ± 1.5	12 ± 2.9	58 ± 5.5	52 ± 5.3	73	<0.001	NS	***	***	***	***	NS
Connectedness	% score	2.2 ± 2.2	27 ± 5.2	55 ± 6.0	56 ± 5.1	40	<0.001	***	***	***	***	***	NS
Closeness with guide	% score	2.5 ± 2.5	33 ± 7.0	39 ± 7.3	43 ± 6.9	11	<0.001	***	***	***	NS	NS	NS
Distressing Experience	% score	0.3 ± 0.3	2.8 ± 1.2	21 ± 4.4	18 ± 5.1	13	<0.001	NS	***	**	***	**	NS
Mystical Experiences Questionnaire (MEQ43)													
Internal unity	% score	0.8 ± 0.8	9.2 ± 3.9	44 ± 5.7	44 ± 5.8	32	<0.001	NS	***	***	***	***	NS
External unity	% score	1.1 ± 1.1	7.5 ± 3.3	43 ± 5.3	40 ± 6.0	33	<0.001	NS	***	***	***	***	NS
Sacredness	% score	1.3 ± 1.3	9.2 ± 3.9	43 ± 5.1	38 ± 5.9	29	<0.001	NS	***	***	***	***	NS
Noetic quality	% score	0.6 ± 0.6	10 ± 3.9	48 ± 5.9	49 ± 6.6	35	<0.001	NS	***	***	***	***	NS
Deeply felt positive mood	% score	1.8 ± 1.8	34 ± 5.2	54 ± 5.9	59 ± 5.2	41	<0.001	***	***	***	**	***	NS
Transcendence of time/space	% score	2.1 ± 2.1	9.3 ± 3.3	59 ± 5.1	53 ± 4.9	78	<0.001	NS	***	***	***	***	NS
Ineffability	% score	2.3 ± 1.3	15 ± 4.1	59 ± 4.5	59 ± 5.5	78	<0.001	NS	***	***	***	***	NS

*P<0.05, **P<0.01, ***P<0.001; NS, not significant; N=24

Table S4. Mean values and statistics for the acute subjective effects on the Adjective Mood Rating Scale (AMRS)

		Placebo mean±SEM	MDMA mean±SEM	LSD mean±SEM	LSD+MDMA mean±SEM	$F_{3,69}$	P=	Pla - MDMA	Pla - LSD	Pla - MDMA + LSD	MDMA - LSD	MDMA - MDMA + LSD	LSD - MDMA + LSD
Adjective Mood Rating Scale (AMRS, score)													
General well-being	ΔE_{max}	1.1 ± 0.6	3.6 ± 1.1	4.9 ± 1.0	4.4 ± 0.9	3.8	0.014	NS	*	*	NS	NS	NS
Emotional excitation	ΔE_{max}	-1.7 ± 0.6	0.3 ± 0.7	5.3 ± 1.0	4.5 ± 1.1	20	<0.001	NS	***	***	***	**	NS
Extraversion	ΔE_{max}	0.5 ± 0.3	2.3 ± 0.5	1.4 ± 0.4	1.6 ± 0.6	2.5	0.071	*	NS	NS	NS	NS	NS
Introversion	ΔE_{max}	0.1 ± 0.3	1.9 ± 0.3	4.8 ± 0.6	5.1 ± 0.5	33	<0.001	*	***	***	***	***	NS
Anxiety	ΔE_{max}	-0.3 ± 0.2	0 ± 0.3	1.0 ± 0.3	1.1 ± 0.3	7.7	<0.001	NS	**	**	*	*	NS
Depression	ΔE_{max}	-0.3 ± 0.1	0.6 ± 0.3	1.6 ± 0.4	1.5 ± 0.3	13	<0.001	NS	***	***	*	*	NS
Activity/Performance	ΔE_{max}	2.0 ± 0.7	1.9 ± 0.8	3.2 ± 1.1	2.0 ± 1.0	0.6	NS	-	-	-	-	-	-
Inactivity	ΔE_{max}	1.8 ± 0.9	6.7 ± 0.9	9.5 ± 1.2	9.8 ± 1.3	15	<0.001	**	***	***	NS	*	NS

*P<0.05, **P<0.01, ***P<0.001; NS, not significant; ΔE_{max}, maximal effect difference from baseline; ΔE_{min}, minimal effect difference from baseline; N=24

Table S5. Parameters characterizing the subjective drug effect-time curves of LSD, MDMA and LSD+MDMA

	MDMA	LSD	LSD+MDMA	$F_{2,46}$	P=
Time to onset (h)	0.5 ± 0.1<br>(0.1 - 1.3)	0.4 ± 0.1<br>(0.1 - 1.1)	0.4 ± 0.1<br>(0.1 - 1.1)	2.2	0.117
Time to offset (h)	4.9 ± 0.4<br>(1.0 - 11)	8.9 ± 0.4***<br>(5.7 - 12)	10 ± 0.8***<br>(5.7 - 21)	46	<0.001
Time to maximal effect (h)	1.9 ± 0.1<br>(1.0 - 3.0)	2.2 ± 0.1<br>(1.0 - 3.5)	2.3 ± 0.3<br>(0.5 - 7.0)	1.4	0.264
Effect duration (h)	4.3 ± 0.4<br>(0.7 - 10)	8.4 ± 0.4***<br>(5.1 - 11)	9.9 ± 0.8***#<br>(5.1 - 21)	49	<0.001
Maximal effect (%)	64 ± 5.6<br>(16 - 100)	90 ± 3.1***<br>(41 - 100)	93 ± 2.6***<br>(47 - 100)	24	<0.001
AUEC	182 ± 28<br>(16 - 635)	494 ± 33***<br>(115 - 786)	575 ± 46***<br>(162 - 1121)	74	<0.001

Parameters are for "any drug effects". The threshold to determine times to onset and offset was set individually at 10% of the individual, maximal response. Values are mean ± SEM (range). *p<0.05, **p<0.01, ***p<0.001 compared with 100 mg of MDMA; #P<0.05 compared with 100 µg LSD ; AUEC, area under the effect curve; N=24

Table S6. Acute and subacute adverse drug effects

	Placebo			MDMA			LSD			LSD+MDMA		
	0h	0-12h	12-24h	0h	0-12h	12-24h	0h	0-12h	12-24h	0h	0-12h	12-24h
Tiredness	12	17	11	13	19	18	13	19	19	12	19	18
Headache	2	11	9	4	16	15	0	17	13	0	16	13
Lack of energy	3	3	4	1	12	7	1	12	10	1	15	9
Loss of appetite	1	1	0	2	13	3	1	12	4	0	16	12
Dry mouth	4	3	1	1	12	0	1	17	5	0	17	6
Lack of concentration	0	2	1	0	16	4	0	6	5	0	16	8
Dullness	1	2	2	1	7	7	1	9	8	2	12	8
Nausea	0	4	1	0	14	2	0	7	2	0	11	3
Brooding	2	1	1	1	11	5	3	4	3	1	11	7
Rapid exhaustibility	0	1	0	1	9	3	0	5	4	0	10	6
Teeth grinding, jaw rigidity	0	1	0	0	9	1	0	11	0	0	14	2
Trembling	0	1	0	0	15	1	0	5	0	0	14	1
Inner restlessness	2	2	0	1	12	2	1	5	0	2	11	2
Feeling of weakness	1	1	0	0	11	4	0	3	1	2	8	5
Restlessness in legs	0	2	0	0	9	1	0	6	1	0	12	2
Inner tension	1	2	0	1	14	2	3	2	0	1	10	2
Excessive perspiration	1	1	1	0	7	3	0	9	1	0	7	2
Heart palpitations	0	2	0	0	9	0	0	9	0	0	10	0
Frequent urination	1	5	0	0	9	2	1	4	1	0	7	1
Hypersensitivity to certain odors	0	0	0	0	9	2	0	2	1	0	6	7

Data indicate number of subjects reporting an effect among a total of 24 subjects.

Table S7. Pharmacokinetic parameters of the metabolites based on non-compartmental analyses [geometric mean (95% CI), range], N=24.

	C_{max} (ng/mL)	t_{max} (h)	$t_{1/2}$ (h)	AUC_{24} (ng·h/mL)	AUC_{∞} (ng·h/mL)	CL/F (L/h)	V_z/F (L)
**O-H-LSD**							
LSD+Placebo administration	0.1 (0.1-0.2)<br>0.01-0.2	5.3 (4.8-5.9)<br>3.5-8.0	8.7 (7.6-9.9)<br>5.6-29	1.8 (1.6-2.1)<br>0.8-3.5	2.4 (2.0-2.7)<br>0.9-5.1	43 (36-50)<br>20-106	533 (466-610)<br>341-1184
LSD+MDMA administration	0.2 (0.1-0.2)<br>0.1-0.3	5.4 (4.7-6.2)<br>3.0-10	10 (8.9-12)<br>5.2-28	2.5 (2.3-2.7)<br>1.6-4.1	3.3 (3.0-3.7)<br>1.9-6.5	30 (27-34)<br>15-52	450 (393-514)<br>246-796
**MDA**							
MDMA+Placebo administration	*11 (9.8-13)<br>7.0-19	*8.0 (7.3-8.8)<br>5.0-12	**14 (12-16)<br>8.6-22	*183 (160-208)<br>114-337	**287 (248-333)<br>165-559	**348 (301-403)<br>179-607	**7057 (6090-8177)<br>3841-12670
LSD+MDMA administration	11 (9.8-13)<br>4.7-18	8.8 (7.9-9.7)<br>4.0-12	*13 (11-14)<br>8.7-34	181 (159-206)<br>78-341	*275 (232-324)<br>109-950	*364 (308-430)<br>105-917	*6700 (5841-7684)<br>4689-14599
**HMMA**							
MDMA+Placebo administration	142 (116-175)<br>33-282	2.8 (2.3-3.3)<br>1.5-6.0	*14 (12-16)<br>10-35	*1740 (1435-2110)<br>437-3038	*2482 (2063-2985)<br>655-4293	*40 (33-48)<br>23-153	*805 (649-998)<br>404-3407
LSD+MDMA administration	132 (110-159)<br>39-252	2.6 (2.3-3.0)<br>1.5-5.0	14 (13-15)<br>7.8-25	1663 (1415-1955)<br>564-2810	2375 (2049-2753)<br>789-4006	42 (36-49)<br>25-127	839 (694-1014)<br>402-2377

AUC, area under the plasma concentration-time curve; AUC_{∞}, AUC from time zero to infinity; AUC_{24}, from time 0-24; CL/F apparent total clearance; C_{max}, maximum observed plasma concentration; $T_{1/2}$, plasma half-life; T_{max}, time to reach C_{max}; 95%CI, 95% confidence interval; V_z/F, apparent volume of distribution; *N=23; **N=22

Table S8. Drug dose identification during the session (2.5h after drug administration), after the session (24h), and after study completion

	Placebo			MDMA			LSD			LSD+MDMA		
	during Session	after Session	after Study	during Session	after Session	after Study	during Session	after Session	after Study	during Session	after Session	after Study
correctly identified	96%	96%	100%	83%	96%	100%	71%	58%	75%	37.5%	46%	75%
missclassified as placebo				0%	0%	0%	0%	0%	0%	0%	0%	0%
missclassified as 100 mg MDMA	4%	4%	0%				4%	4%	0%	12.5%	8%	0%
missclassified as 100 µg LSD	0%	0%	0%	4%	0%	0%				50%	46%	25%
missclassified as 100 mg MDMA + 100 µg LSD	0%	0%	0%	13%	4%	0%	25%	38%	25%			

References

1 Holze F, Vizeli P, Muller F, Ley L, Duerig R, Varghese N, et al. Distinct acute effects of LSD, MDMA, and D-amphetamine in healthy subjects. Neuropsychopharmacology. 2020;45(3):462-71.

2 Schmid Y, Enzler F, Gasser P, Grouzmann E, Preller KH, Vollenweider FX, et al. Acute effects of lysergic acid diethylamide in healthy subjects. Biol Psychiatry. 2015;78(8):544-53.

3 Hysek CM, Schmid Y, Simmler LD, Domes G, Heinrichs M, Eisenegger C, et al. MDMA enhances emotional empathy and prosocial behavior. Soc Cogn Aff Neurosci. 2014;9:1645-52.

4 Holze F, Duthaler U, Vizeli P, Muller F, Borgwardt S, Liechti ME. Pharmacokinetics and subjective effects of a novel oral LSD formulation in healthy subjects. B J Clin Pharmacol. 2019;85:1474-83.

5 Dolder PC, Schmid Y, Steuer AE, Kraemer T, Rentsch KM, Hammann F, et al. Pharmacokinetics and pharmacodynamics of lysergic acid diethylamide in healthy subjects. Clin Pharmacokinetics. 2017;56:1219-30.

6 Dolder PC, Strajhar P, Vizeli P, Hammann F, Odermatt A, Liechti ME. Pharmacokinetics and pharmacodynamics of lisdexamfetamine compared with D-amphetamine in healthy subjects. Front Pharmacol 2017;8:617.

7 Tagliazucchi E, Roseman L, Kaelen M, Orban C, Muthukumaraswamy SD, Murphy K, et al. Increased global functional connectivity correlates with LSD-induced ego dissolution. Curr Biol. 2016;26(8):1043-50.

8 Liechti ME, Dolder PC, Schmid Y. Alterations in conciousness and mystical-type experiences after acute LSD in humans. Psychopharmacology. 2017;234:1499-510.

9 Janke W, Debus G. Die Eigenschaftswörterliste. Hogrefe: Göttingen.; 1978.

10 Dittrich A. The standardized psychometric assessment of altered states of consciousness (ASCs) in humans. Pharmacopsychiatry. 1998;31 (Suppl 2):80-4.

11 Studerus E, Gamma A, Vollenweider FX. Psychometric evaluation of the altered states of consciousness rating scale (OAV). PLoS One. 2010;5(8):e12412.

12 Carhart-Harris RL, Kaelen M, Bolstridge M, Williams TM, Williams LT, Underwood R, et al. The paradoxical psychological effects of lysergic acid diethylamide (LSD). Psychol Med. 2016;46:1379-90.

13 Dolder PC, Schmid Y, Mueller F, Borgwardt S, Liechti ME. LSD acutely impairs fear recognition and enhances emotional empathy and sociality. Neuropsychopharmacology. 2016;41:2638-46.

14 Bershad AK, Schepers ST, Bremmer MP, Lee R, de Wit H. Acute subjective and behavioral effects of microdoses of lysergic acid diethylamide in healthy human volunteers. Biol Psychiatry. 2019;86(10):792-800.

15 Preller KH, Herdener M, Pokorny T, Planzer A, Kraehenmann R, Stämpfli P, et al. The fabric of meaning and subjective effects in LSD-induced states depend on serotonin 2A receptor activation Curr Biol. 2017;27:451-57.

16 Schmid Y, Gasser P, Oehen P, Liechti ME. Acute subjective effects in LSD- and MDMA-assisted psychotherapy. J Psychopharmacol. 2021;35(4):362-74.

17 Roseman L, Nutt DJ, Carhart-Harris RL. Quality of acute psychedelic experience predicts therapeutic efficacy of psilocybin for treatment-resistant depression. Front Pharmacol. 2017;8:974.

18 Griffiths RR, Johnson MW, Carducci MA, Umbricht A, Richards WA, Richards BD, et al. Psilocybin produces substantial and sustained decreases in depression and anxiety in patients with life-threatening cancer: a randomized double-blind trial. J Psychopharmacol. 2016;30(12):1181-97.

19 Griffiths RR, Richards WA, McCann U, Jesse R. Psilocybin can occasion mystical-type experiences having substantial and sustained personal meaning and spiritual significance. Psychopharmacology (Berl). 2006;187(3):268-83; discussion 84-92.

20 Barrett FS, Johnson MW, Griffiths RR. Validation of the revised Mystical Experience Questionnaire in experimental sessions with psilocybin. J Psychopharmacol. 2015;29(11):1182-90.
21 MacLean KA, Johnson MW, Griffiths RR. Mystical experiences occasioned by the hallucinogen psilocybin lead to increases in the personality domain of openness. J Psychopharmacol. 2011;25(11):1453-61.
22 Griffiths RR, Johnson MW, Richards WA, Richards BD, McCann U, Jesse R. Psilocybin occasioned mystical-type experiences: immediate and persisting dose-related effects. Psychopharmacology. 2011;218(4):649-65.
23 Griffiths R, Richards W, Johnson M, McCann U, Jesse R. Mystical-type experiences occasioned by psilocybin mediate the attribution of personal meaning and spiritual significance 14 months later. J Psychopharmacol. 2008;22(6):621-32.
24 Garcia-Romeu A, Griffiths RR, Johnson MW. Psilocybin-occasioned mystical experiences in the treatment of tobacco addiction. Curr Drug Abuse Rev. 2014;7(3):157-64.
25 Garcia-Romeu A, Davis AK, Erowid F, Erowid E, Griffiths RR, Johnson MW. Cessation and reduction in alcohol consumption and misuse after psychedelic use. J Psychopharmacol. 2019;33(9):1088-101.
26 Griffiths RR, Johnson MW, Richards WA, Richards BD, Jesse R, MacLean KA, et al. Psilocybin-occasioned mystical-type experience in combination with meditation and other spiritual practices produces enduring positive changes in psychological functioning and in trait measures of prosocial attitudes and behaviors. J Psychopharmacol. 2018;32:49-69.
27 Ross S, Bossis A, Guss J, Agin-Liebes G, Malone T, Cohen B, et al. Rapid and sustained symptom reduction following psilocybin treatment for anxiety and depression in patients with life-threatening cancer: a randomized controlled trial. J Psychopharmacol. 2016;30(12):1165-80.
