# Supplementary material for: Acute effects of MDMA and LSD co-administration in a double-blind placebo-controlled study in healthy participants
Source: Neuropsychopharmacology. 2023 May 31;48(13):1840–8. doi: 10.1038/s41386-023-01609-0 (PMC10584820; doi:10.1038/s41386-023-01609-0)
Supplement: Supplementary file 2 — CONSORT Flowchart [file 41386_2023_1609_MOESM2_ESM.pdf]

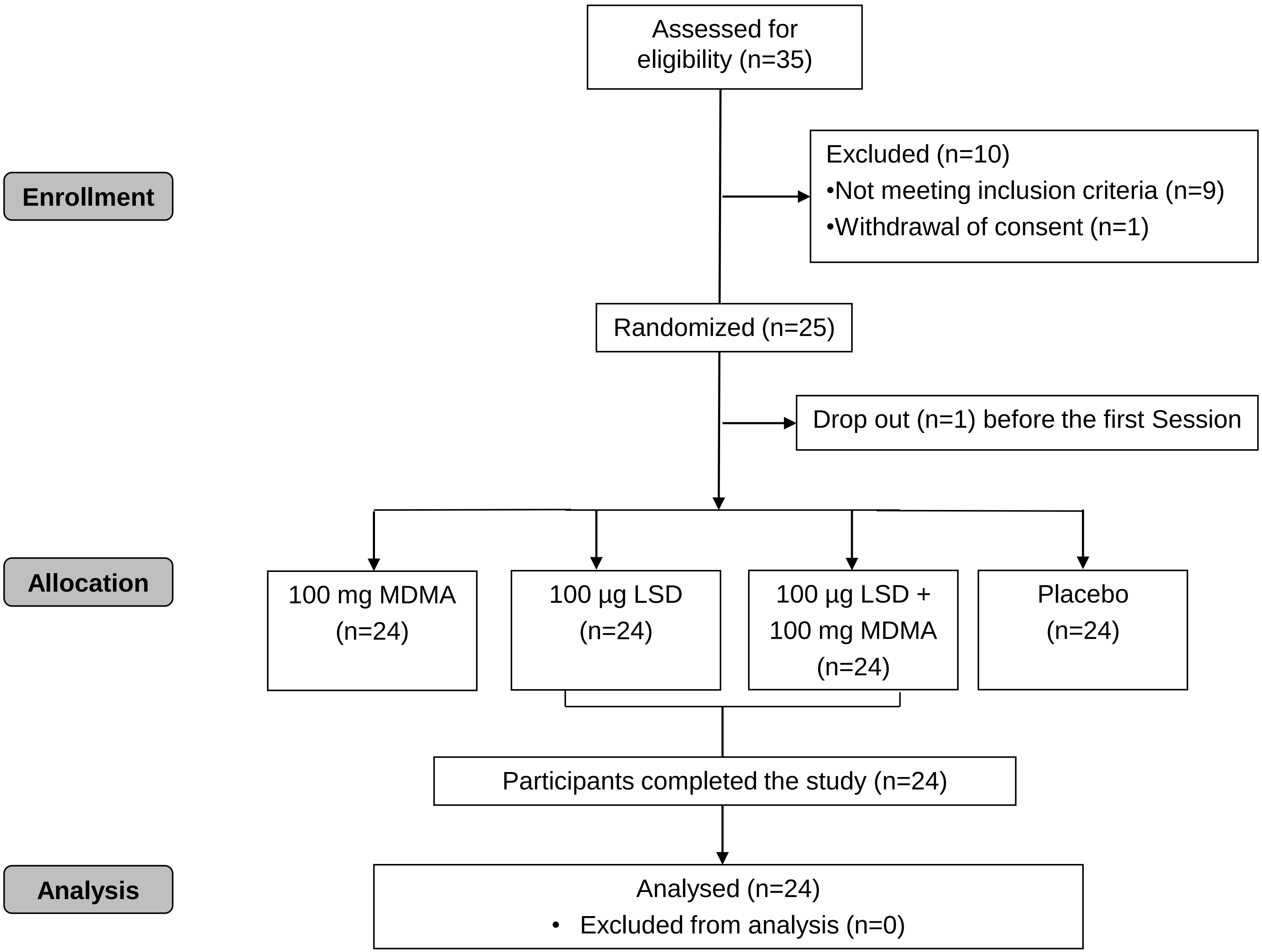
Assessed for eligibility (n=35)
Enrollment
Excluded (n=10)
•Not meeting inclusion criteria (n=9)
•Withdrawal of consent (n=1)
Randomized (n=25)
Drop out (n=1) before the first Session
Allocation
100 mg MDMA (n=24)
100 µg LSD (n=24)
100 µg LSD + 100 mg MDMA (n=24)
Placebo (n=24)
Participants completed the study (n=24)
Analysis
Analysed (n=24)
• Excluded from analysis (n=0)
